# Effectiveness of Sensitization Campaigns in Reducing Leprosy-Related Stigma in Rural Togo: Protocol for a Mixed Methods Cluster Randomized Controlled Trial

**DOI:** 10.2196/52106

**Published:** 2024-04-18

**Authors:** Dominik Jockers, Akila Wimima Bakoubayi, Kate Bärnighausen, P'tanam P'kontème Bando, Stefanie Pechar, Teresia Wamuyu Maina, Jonas Wachinger, Mark Vetter, Yawovi Djakpa, Bayaki Saka, Piham Gnossike, Nora Maike Schröder, Shuyan Liu, Denis Agbenyigan Yawovi Gadah, Christa Kasang, Till Bärnighausen

**Affiliations:** 1 Heidelberg Insititute of Global Health Faculty of Medicine and University Hospital Heidelberg University Heidelberg Germany; 2 German Leprosy and Tuberculosis Relief Association Lomé Togo; 3 Public Health and Prevention Technical University of Munich Munich Germany; 4 Geovisualization Technical University of Applied Sciences Würzburg-Schweinfurt Würzburg Germany; 5 Department of Dermatology Tsévié Hospital University of Lomé Lomé Togo; 6 National Programme for Neglected Tropical Diseases Lomé Togo; 7 Department of Psychiatry and Psychotherapy (Campus Charité Mitte) Charité – Universitätsmedizin Berlin Berlin Germany; 8 German Leprosy and Tuberculosis Relief Association Würzburg Germany

**Keywords:** audio, community health worker, information campaign, knowledge, attitude, and practices, language, leprosy-related stigma, qualitative and quantitative research, stigma intervention, Togo, West Africa

## Abstract

**Background:**

In the global strategy to eliminate leprosy, there remains a need for early case detection to successfully interrupt transmissions. Poor knowledge about leprosy and leprosy-related stigma are key drivers of delayed diagnosis and treatment. Sensitization campaigns to inform and increase awareness among the general population are an integral part of many national neglected tropical disease programs. Despite their importance, the effectiveness of such campaigns has not been rigorously studied in the West African context. A multilingual rural setting with low health literacy in this region presents challenges to the potential impact of sensitization campaigns.

**Objective:**

The primary objective of this study is to assess the causal effect of common practice community sensitization campaigns on leprosy-related knowledge and stigma at the community level and among community health volunteers. Additionally, we will test the potential of novel educational audio tools in the 15 most prominent local languages to overcome literacy and language barriers and amplify sensitization campaigns.

**Methods:**

We will conduct a cluster randomized controlled trial using a sequential mixed methods approach in 60 rural communities across all regions of Togo, West Africa. The study features 2 intervention arms and 1 control arm, with intervention and control assignments made at the community level through randomization. Communities in intervention arm 1 will receive a sensitization campaign in line with the current Togolese national neglected tropical disease program. Communities in intervention arm 2 will receive the same sensitization campaign along with educational audio tools distributed to community households. The control arm will receive no intervention before data collection. Quantitative outcome measures on knowledge and stigma will be collected from a random sample of 1200 individuals. Knowledge will be assessed using the 9-item standardized Knowledge, Attitudes, and Practices Questionnaire. Stigma will be measured using the 7-item Social Distance Scale and the 15-item Explanatory Model Interview Catalogue Community Stigma Scale. We will estimate intention-to-treat effects at the individual level, comparing the outcomes of the intervention and control arms. In an accompanying qualitative component, we will conduct in-depth interviews with community members, community health volunteers, and health care workers in both treatment arms and the control arm to explore intervention and stigma-related experiences.

**Results:**

This paper describes and discusses the protocol for a mixed methods cluster randomized controlled trial. Data collection is planned to be completed in June 2024, with ongoing data analysis. The first results are expected to be submitted for publication by the end of 2024.

**Conclusions:**

This trial will be among the first to test the causal effectiveness of community-based sensitization campaigns and audio tools to increase knowledge and reduce leprosy-related stigma. As such, the results will inform health policy makers, decision-makers, and public health practitioners designing sensitization campaigns in rural multilingual settings.

**Trial Registration:**

German Clinical Trials Register DRKS00029355; https://drks.de/search/en/trial/DRKS00029355

**International Registered Report Identifier (IRRID):**

DERR1-10.2196/52106

## Introduction

### Overview

Leprosy is a communicable neglected tropical disease (NTD) caused by *Mycobacterium leprae*. In 2022, more than 140,000 cases were reported globally, mainly in Southeast Asia, Africa, and the Americas [1]. Across sub-Saharan African countries, leprosy is found in endemic pockets and is most common among the rural poor [2,3]. Despite effective antibiotic treatment being widely available, new cases of leprosy continue to occur in Togo [4]. In line with experiences in other countries, a significant number of hidden cases is expected [1,5,6]. In its global strategy to eliminate leprosy, the World Health Organization emphasizes the need for early case detection to successfully interrupt transmissions. Additionally, as an independent objective, the need to reduce stigma is emphasized to further alleviate the burden of the disease [7].

Leprosy is a highly stigmatized disease, mainly due to low understanding and knowledge about the disease itself, including misconceptions regarding transmission and treatment [8-10]. While knowledge and stigma are distinct constructs, they often interact. Consequently, a lack of knowledge about the disease can foster false beliefs that may contribute to the stigmatization of patients. However, even with increased understanding of the disease, stigmatizing attitudes can persist [8]. This stigma contributes to patients’ hesitation to seek care [10], as well as delayed diagnosis and treatment [11,12]. As a consequence, a delayed or missed diagnosis of leprosy often results in irreversible disabilities causing permanent visible impairments [13], which in turn reinforce stigma and discrimination [14].

Existing sensitization campaigns aim to create awareness and improve understanding of leprosy among the general population and ultimately achieve early case detection and treatment [15]. Common practice leprosy sensitization campaigns include information on the disease itself, transmission, incubation, development, and potential treatment. Group discussions, leaflets, and positive testimonies of people successfully cured are used to overcome potential entrenched misconceptions among the population. Although there is a comprehensive body of literature on conceptualizing the drivers and consequences of health-related stigma [8,16-20], the effectiveness assessment of interventions targeting leprosy-related stigma are rare. A nonrandomized intervention study in India found health posters and focus group discussions to be associated with improved leprosy-related knowledge and reduced stigma [21]. A before-and-after study in Indonesia also showed leprosy sensitization campaigns to be associated with a reduction of stigma [22]. To the best of our knowledge, the effectiveness of leprosy-related stigma interventions has not been studied in the West African context.

Togo has a literacy rate of 67%, and aside from French being the official working language, 49 local languages are widely spoken [23]. Especially in rural communities, local languages are often the predominant mother tongue. Several scholars have shown the impact of language on education outcomes in the multilingual sub-Saharan African context [24-26]. As such, literacy and language barriers are challenging to the potential effectiveness of one-time sensitization campaigns to build comprehensive knowledge and reduce stigma.

In this study, we assess the effectiveness of community-based sensitization campaigns in increasing leprosy-related knowledge and reducing stigma among the target populations in rural Togo. The common practice sensitization campaigns are carried out by the Togolese National NTD program. The campaigns are building on the inclusion of local health care workers (HCWs) and community health volunteers (CHVs) to mobilize community members and convey knowledge. Furthermore, we will establish the effectiveness of novel audio-based tools that provide target populations with information in local languages to overcome language barriers and increase the effectiveness of sensitization campaigns.

We will conduct a mixed methods cluster randomized controlled trial (cRCT) study in 60 rural communities in Togo.

### Study Objectives

The main objective of this study is to assess the effectiveness of community-based sensitization campaigns to increase leprosy-related knowledge and reduce stigma. Our results will inform national policy and decision makers, as well as stakeholders delivering health services and sensitization campaigns.

Specific research objectives are to (1) establish the effectiveness of community-level sensitization campaigns and local language audio tools to increase knowledge and decrease stigma related to leprosy among community members; (2) determine the effects of leprosy training for CHVs (knowledge on diagnosis, treatment, and prevention); and (3) elucidate the mechanisms and experiences of sensitization interventions among community members, HCWs, and CHVs.

### Trial Design

We will use a cRCT using a sequential mixed methods design by collecting and analyzing quantitative and qualitative data. The trial will have 2 intervention arms that are randomly assigned across 60 study communities. Intervention arm 1 will receive the sensitization campaign (common practice by the national NTD program). Intervention arm 2 will receive the sensitization campaign (a common practice by the national NTD program) accompanied by audio tools. Control arm will receive no sensitization campaign exposure before endline data collection.

Communities assigned to the control arm will receive intervention arm 1 after data collection to ensure that all communities will receive common practice sensitization campaigns by the end of the study period.

### Conceptual Framework

We use the universal health-related stigma and discrimination framework proposed by Stangl et al [27] to guide our understanding of leprosy-related stigma. Stigma is understood as an attribute discrediting the patient, enabling discrimination, and eventually limiting the opportunities of the affected persons [16,18]. The stigma and discrimination framework describes 4 different stages of health-related stigma: the first stage includes drivers and facilitators of stigma, followed by stigma marking to either health conditions or characteristics of affected groups in the second stage. Once stigma marking takes place, the third phase represents the manifestation of stigma, with experience and anticipation of stigma and stigmatizing behavior. Eventually, the manifestation of stigma affects several outcomes in the fourth stage at the individual level for affected persons and at the institutional level, manifesting as discriminating laws and guidelines. Following Stangl et al [27], the first 3 stages are key areas of applied research and interventions aiming at health-related stigma reduction.

In the context of leprosy, potential drivers of stigma are the fear of infection, social exclusion, and devaluation [8]. Misconceptions of leprosy, such as infection being a consequence of committed sins or social misconduct, can also drive stigma [28,29]. Facilitators of stigma are characteristics of affected groups such as low socioeconomic status or low educational background [8,30]. Leprosy-related stigma is commonly expressed by the loss of the social status, reputation, and self-esteem of affected persons. Patients are often socially isolated and face difficulties achieving socially desirable goals such as marriage [31]. Stigma manifestation also includes the anticipation and internalization of stigma [20]. As a consequence of stigma, early symptoms are often concealed, and delays in diagnosis lead to treatment being initiated at a disease stage where permanent, visible impairments and irreversible disabilities already occur.

While we acknowledge that education alone is never as effective as a combination of strategies for stigma reduction [8,32,33], the interventions in this study are designed to primarily target underlying causes and drivers of stigma by addressing knowledge and beliefs about leprosy at the community level. Additionally, the interventions also engage community members in activities aimed directly at reducing stigma. Through the inclusion of audio tools for information dissemination, we aim to acknowledge potential stigma facilitators, such as low literacy rates, in affected groups [30]. Offering learning content in local languages (rather than French), the audio tools could potentially improve campaign effectiveness by increasing self-efficacy through the ability to participate in the intervention as well as being able to understand the content [24-26]. Additionally, our trainings for CHVs and health facility staff in our intervention arms aim to reduce stigma at the organizational and institutional level (for example, avoiding segregation of patients) [15].

## Methods

### Study Setting and Implementation Partner

This study will be conducted in rural communities in Togo, West Africa. Study communities have been selected from the national NTD database. A community is considered eligible if it is located in a rural area and if at least one leprosy case has been reported in the national register between 2010 and 2020. A community has been classified as rural if its population (aged 18 years and older) did not exceed 1500 individuals based on remote censoring data [34]. Selected study sites span all 6 regions of Togo, yet districts bordering Burkina Faso were excluded due to security concerns. Between 2010 and 2014, 2630 new cases of leprosy were recorded by the Togolese National Leprosy/Buruli Ulcer Control Program. Nonetheless, a high number of undetected cases are expected [5,6]. To increase awareness, promote active case finding, and facilitate access to leprosy treatment, the Togolese National NTD program is working closely with the international nongovernment organization German Leprosy and Tuberculosis Relief Association (DAHW). The main activities of the collaboration include the implementation of skin screening campaigns in particularly vulnerable communities at risk of leprosy. During campaigns, individuals can receive professional skin screenings by dermatologists and appropriate treatment if needed. To increase help-seeking and prevent stigmatization of diagnosed patients, skin screening camps are preceded by a sensitization campaign on general skin diseases and leprosy in particular. Mobilization for sensitization campaigns and skin screenings is typically done by the respective HCWs and CHVs. Where HCWs are trained staff working from health facilities, CHVs received basic training and are voluntarily providing health services within their communities. The sensitization campaigns implemented by the National NTD program and the DAHW form the basis of the intervention to be evaluated in this study, and study team members include representatives of the Togolese National NTD program and the DAHW to facilitate data collection and implementation.

### Intervention Description

The sensitization campaigns in intervention arms 1 and 2 will be carried out in three steps: (1) planning, (2) social mobilization, and (3) implementation. The planning of sensitization and skin screening activities will be done in collaboration with influential forces in the community (eg, traditional chiefs, religious leaders, school principals, CHVs). Choices regarding the date, time, and place of the activity will be made in collaboration with the local opinion leaders. The social mobilization will entail the mobilization of all influential forces in the community to encourage individuals and families to participate in the activity.

On the day of the implementation of the sensitization activities, community members will be invited to educational sessions. The CHVs will give key information on skin-related NTDs, including leprosy. HCWs will support the CHVs to ensure the accuracy of the information and message given out. Topics covered will be clinical signs, symptoms, treatment, environmental aspects conducive to the contraction of the disease, methods of prevention, management, and prevention of disabilities. The knowledge dissimilation will be done with the help of posters, images of leprosy, and the screening of a documentary film on skin-related NTDs, followed by community debates. In addition, community members are actively involved in activities aimed at reducing stigmatizing attitudes and behaviors toward individuals affected by leprosy. Community leaders play a pivotal role in developing strategies to transform community norms that contribute to the stigma surrounding leprosy patients.

After the sensitization stage, consultations will take place for the screening of cutaneous dermatoses. In a private area, health staff will individually assess people who presents with a lesions, sores, or stains on the body. First aid will be offered by health professionals to any person who presents with a skin disorder, and individuals diagnosed with leprosy will be referred to the health facility for treatment initiation and follow-up consultations.

In intervention arm 2, in addition to the activities described above, the CHVs will offer audio tools to households in the study communities. The solar powered tools will contain the learning content of the campaign in audio form [35]. The content will be translated into the 15 most prominent local languages of the communities in intervention arm 2. The tools will remain within the community until endline data collection (approximately 2 months) to allow repetitive learning for all household members.

### Implementation

This study is a cRCT, with the randomization unit being the administrative communities. We stratified the random assignment by administrative regions in Togo (Maritime, Plateaux, Central, Kara, and Savanes) to ensure representation of all intervention and control arms across all regions. Since the sensitization campaigns are carried out at the community level and are targeted at the whole community population, cluster randomization was the natural choice of study design. Participation in the sensitization campaign is voluntary, open to all community members, and does not imply participation in the study as such. Survey participants will be recruited from the general community population, irrespective of their participation in the sensitization campaigns.

We used computer-generated random numbers for intervention arm assignment. We set a random seed to 585,506 in Stata (StataCorp) to execute a replicable and random intervention arm assignment. Misfits at the regional level have been balanced at the global level.

Quantitative data have been collected at baseline in all 60 communities and will be complemented with an endline survey 2 months after intervention implementation. Primary outcomes will be collected in all intervention arms before and after intervention.

Baseline data collection took place in March 2023, and endline survey data and qualitative data are planned to be collected in November 2023. Training of health volunteers and professionals and the implementation of campaigns will take place between August and September 2023.

### Eligibility Criteria

As described above, intervention participation is voluntary, open to all community members, and does not imply participation in the survey as such. Eligibility criteria for survey participation vary by study component.

#### Quantitative Study Component

People aged 18 years and older who are resident of the respective community. All CHVs serving a study community and aged 18 years and older will be eligible to participate in the survey.

#### Qualitative Study Component

People aged 18 years and older and are residents of the respective community. We will also purposefully select some patients with leprosy. CHVs and HCWs serving a target study community and aged 18 years and older will be eligible to participate in the study.

### Outcome Measures

#### Quantitative End Points

Our primary outcomes to assess leprosy-related knowledge among community members will be based on the standardized Knowledge, Attitudes, and Practices Questionnaire adapted to leprosy. We will assess different forms and perceptions of leprosy-related stigma using the Social Distance Scale, asking for individuals own stigmatizing attitudes. Further, we will ask about the perceived attitudes and behaviors in the community using the Explanatory Model Interview Catalogue (EMIC). Individuals not affected by leprosy will receive the Community Stigma Scale. Affected individuals will receive questions related to their experience with stigma. In addition, we will assess a number of secondary outcomes, such as the Participation Scale. The primary and secondary outcomes are shown in Textbox 1.

**Primary and secondary outcomes.**

**Primary outcomes**

- 15-item Knowledge, Attitudes and Practices Questionnaire [36].
- 7-item Social Distance Scale [36].
- 15-item Explanatory Model Interview Catalogue Community Stigma Scale for persons not affected and Explanatory Model Interview Catalogue for persons affected [37].

**Secondary outcomes**

- 18-item Participation Scale [38].
- 10-item Rosenberg Self-Esteem Scale [39].
- 3-item University of California, Los Angeles Loneliness Scale [40].
- 2-item Patient Health Questionnaire [41].
- 2-item Generalized Anxiety Disorder Scale [42].
- 3-item Alcohol Use Disorders Identification Test for Consumption [43].

#### Qualitative End Points

We will explore the needs, preferences, and understanding of HCWs and CHVs regarding training on leprosy treatment and care. With in-depth interviews (IDIs) and shared walks, we will build place-based data, examine the practices and perceptions of HCWs and CHVs relating to pathways to treatment and care, and analyze how stigma may mediate these pathways. Additionally, our IDIs with community members will explore cultural norms and beliefs around health seeking and treatment for leprosy and examine perceptions and experiences regarding the sensitization campaign.

Areas of particular interest are (1) understanding the perceptions of HCWs and CHVs regarding the use and appropriateness of the training received on leprosy treatment and care; (2) understanding the perceptions of HCWs and CHVs regarding stigma, diagnosis, treatment, and care of leprosy; and (3) understanding community perceptions regarding the sensitization campaign, the audio tool, and the diagnosis, treatment, and care of leprosy.

### Sample Size

#### Quantitative Sample Size

We conducted a power calculation for our primary end points to determine the required sample size. The power calculation considers the study design of randomized intervention assignment across clusters (communities). To reach a power of 80% in detecting an effect size of 10 percentage points at a significance level of 5% (SD 30, intracluster correlation coefficient=0.05), a sample of a minimum of 15 individuals in each community is given a fixed number of 60 clusters. This leads to an overall required sample size of 900 individuals. To account for potential complications, we targeted a sample size of 1200 individuals. Baseline data collection took place in March 2023, with 1200 individuals successfully interviewed.

#### Qualitative Sample Size

To determine the qualitative sample size, we follow the principle of achieving data saturation, which ensures that reoccurring themes are exhausted [44]. With a study that uses IDIs and shared walks—with a relatively focused research question—saturation should occur at approximately 20 interviews or with 20 participants. Therefore, we aim to conduct 5-10 IDIs with HCWs in each of the intervention and control arms (15-30 in total) and 10-15 IDIs with community members in each of the intervention and control arms (30-45 in total).

### Recruitment and Data Collection

Over the course of the study, we will integrate several rounds of quantitative and qualitative data collection with community members, CHVs, and HCWs. For an overview of all data collection instruments, see Table 1.

**Table 1:** Data collection instruments of a mixed methods cluster randomized controlled trial to assess the effectiveness of leprosy sensitization campaigns in rural Togo. A summary of study instruments, respondents, and procedures. Data collection and intervention implementation will take place between March 2023 and June 2024.

Data collection type and instrument			Population and recruitment												Sample size			Estimated duration (minutes)			Timing of data collection
			Population			Observation unit		Sampling unit		Sampling method		Eligibility									
**Quantitative**																					
	Survey baseline		Community members of all 60 study communities			Individual		Households		Random selection		Adult men and women aged ≥18 years, preferably household head and spouse		1200			20			Before Intervention	
	Survey endline		Community members of all 60 study communities			Individual		Households		Random selection		Adult men and women aged ≥18 years, preferably household head and spouse		1200			30			After Intervention	
	Survey CHV^a^		CHVs of all intervention communities			Individual		Community		Census (intervention arm 1 and 2)		CHV of study community		40			20			After Intervention	
**Qualitative**																					
		In-depth interview cover sheet		HCWs^b^, CHVs and community	Individual		Community		Purposive		Adult men and women aged ≥18 years and HCW, CHV or community member in the respective communities that provide written informed consent		45-75			N/A^c^			During intervention		
		In-depth interview guide		HCWs and CHVs	Individual		Community		Purposive		Adult men and women aged ≥18 years and HCW, CHV or community member in the respective communities that provide written informed consent		15-30			45-60			During intervention		
		Shared walk guide		HCWs and CHVs	Individual		Community		Purposive		Adult men and women aged ≥18 years and HCW, CHV or community member in the respective communities that provide written informed consent		15-20			N/A			During intervention		
		In-depth interview guide		Community members	Individual		Community		Purposive		Adult men and women aged ≥18 years that identify as a community member in the respective communities and provide written informed consent		30-45			45-60			During intervention		

^a^CHV: community health volunteer.

^b^HCW: health care worker.

^c^N/A: not applicable.

#### Quantitative Survey Participants

At baseline, before intervention implementation, community members were sampled through a random selection of households in all 60 study communities. In each community, 10 households were selected following a random walk procedure adjusted to the local context of widely spread farmsteads [45]. In each selected household, one female and one male household member were interviewed, preferably the household head and respective spouse or husband. For the endline survey, the same individuals will be interviewed. All CHVs will be selected for a quantitative survey during the endline data collection.

#### Qualitative Interview and Shared Walk Participants

We will conduct IDIs with 5-10 HCWs and CHVs in each of the intervention and control arms. In this phase, we will also invite selected 15-20 IDI respondents to participate in a “shared walk” to walk through their community and show research assistants (RAs) where their work takes place, what locational influences there are on their work, and how they feel anything influences their work in relation to leprosy. We will also conduct IDIs with community members in each of the intervention and control arms (30-45 members in total) to understand (depending on the intervention assignment) their perceptions of the services being offered, the sensitization campaign, and their feelings and experiences regarding leprosy.

A total of 3 RAs will conduct the IDIs and shared walks using standardized instruments. These include a participant information sheet, a consent form, a cover sheet, and interview guides (Table 1). Cover sheets will capture sociodemographic data, including sex, age, employment status, children, partner status, and religion. Cover sheets also include a section for RAs to make reflexive and observational notes. Written informed consent will be obtained from all study participants before beginning interviews. Depending on the preference of the participants, IDIs will be conducted one-on-one in a local language or French and will be audio-recorded. Participants in the IDIs will be asked broad, open-ended questions regarding topics such as the training, the intervention and audio tool, stigma, and perceptions of leprosy. IDIs will be designed to elicit personal responses and then lead to more in-depth and specific narrative-building questions. RAs will probe themes that seem to be of relevance to the participant or that are identified as important recurring themes through the debriefing process.

The “shared walk” will follow the tenets of the docent method defined by Chang [46] to provide more place-based data. The participants decide the route and duration of the walk. The RA will ask the participant to show them locations on the walk that are of significance to them for any reason, but will also ask the participant to show locations that are of particular relevance for the work they do and where significant events (such as education or outreach) take place. Each participant is considered to be an expert guide. The participant is the educator, while the RA is the person who needs to learn from and follow the lead of the participant. These walks will be audio recorded and can be supported by photos if the participant wishes to take them. Any photographs submitted in which it is possible to identify individuals will be anonymized.

### Data Analysis

#### Quantitative Analysis

Our primary analysis will be based on intention-to-treat at the individual level using linear regression comparing the outcomes of intervention and control arms. We will measure our primary outcomes in terms of absolute scores and as a percentage of the total possible score. We will use adjusted standard errors for clustering at the community level, our unit of randomization [47]. We will control for a number of baseline covariates, including gender, age, and education, among others.

#### Qualitative Analysis

Analysis will begin in the field with systematic debriefings. Debriefings are daily meetings with RAs and are designed to identify where interview questions are gaining in-depth responses and where questions need to be changed or refined [48]. Debriefings will also allow the research team to gain a superficial understanding of the main topics arising from the interviews and the shared walks, so that an initial codebook can be developed [48]. Once all data have been collected, IDIs will be translated, transcribed, and managed using NVivo (version pro 12; QSR International) [49]. We will follow a reflexive thematic analysis approach using the recursive 6 stages of analysis as defined by Braun and Clarke [50]. Data analysts will independently read and re-read transcripts for data familiarization. Transcripts will then be inductively analyzed in blocks of 5, and codes will be reviewed to identify similarities or divergence of ideas. This process will be followed until the coding is complete. The main codes will be presented to the study team to decide where reoccurring codes could build our core themes. After revising the codebook, we will develop a thematic scheme that will be presented to the study team, refined, named, and finalized for interpretation and writing.

Our mixed methods process will follow a sequential approach where we begin with quantitative data collection and analysis and, upon completion, start the qualitative component [51]. We assume that the quantitative data will allow us to develop more focused qualitative questions and that the qualitative data will help us to address any gaps in information that we cannot explain through the quantitative results. Further mixing of the 2 methods will occur during the data analysis of both data sets, when we will combine the qualitative and quantitative to present our findings to support the interpretation of the results. As such, we envision the shared walk data to allow us to identify potential intervention improvements with respect to the information provided, the dissemination of this information, and, in particular, participant understanding of the audio tool.

### Ethical Considerations

This study was approved by the Togolese Bioethics Committee for Health Research (025/2022) and the Ethics Commission of the Medical Faculty Heidelberg (S-670/2022), Germany. Before consent, information about the study will be provided (verbal or written). All participants will give (verbal or written) informed consent to participate in the research. Participants will not receive compensation for their participation. The data will be deidentified before analysis and securely stored in a password-protected file and on a password-protected computer. Any photographs submitted during qualitative data collection, in which it is possible to identify individuals, will be anonymized. The trial is registered at the German Clinical Trials Register (DRKS-ID: DRKS00029355).

## Results

Data collection started in March 2023 and is planned to be completed in June 2024, with ongoing data analysis. Analysis of the quantitative baseline data has been initiated, and results are planned to be submitted for peer-reviewed publication in the second quarter of 2024. The first quantitative endline and qualitative results are expected to be submitted for publication by the end of 2024.

## Discussion

We will use the mixed methods cRCT to measure the causal effect of community sensitization campaigns and audio tools distributed to households on knowledge and stigma related to leprosy. The setting of this study is rural communities in Togo, West Africa. Our nested qualitative components, involving interviews and shared walks with community members and local stakeholders such as CHVs and HCWs, will explore the implementation of the intervention and contextualize the quantitative results of the trial, while also helping to further characterize the mechanisms through which sensitization campaigns and audio tools affect leprosy-related knowledge and stigma.

The contribution of this study to the literature will be 3-fold. First, we will provide causal evidence on the effectiveness of sensitization campaigns on community-level leprosy-related knowledge and stigma. The literature on this is limited, especially in the West African context. Second, we will test novel audio tools for information dissemination to amplify potential campaign effects. The tools are designed to overcome literacy and language barriers in a multilingual context. Third, our results will contribute to the general understanding of leprosy-related stigma in the West African context. The qualitative and quantitative findings will inform policy makers and public health agents to effectively tailor and implement sensitization campaigns, particularly in challenging rural settings.

A potential limitation of this study is the fact that quantitative stigma indicators are self-reported by community members; this outcome might be affected by social-desirability bias. We will test intervention effects on the respondent’s knowledge of the disease, which is a key driver of stigma and not prone to social desirability bias. Further, qualitative IDIs with unaffected and affected community members will allow us to triangulate quantitative findings on stigma.

This cRCT serves to provide rigorous scientific evidence about the causal effectiveness of 2 particular interventions in the broader field of health-related stigma interventions and multilingual low-literacy settings. The results will be beneficial for policy makers and public health agents to guide sensitization campaigns and inform them about the potential of audio tools to complement common practice.

## Abbreviations

CHV: community health volunteer
cRCT: cluster randomized controlled trial
DAHW: German Leprosy and Tuberculosis Relief Association
EMIC: Explanatory Model Interview Catalogue
HCW: health care worker
IDI: in-depth interview
NTD: neglected tropical disease
RA: research assistant

## Data Availability

The final data set used and analyzed for this study will be available from the corresponding author on reasonable request.
